# Phenotype and genotype in patients with Larsen syndrome: clinical homogeneity and allelic heterogeneity in seven patients

**DOI:** 10.1186/s12881-016-0290-6

**Published:** 2016-04-06

**Authors:** Katta Mohan Girisha, Abdul Mueed Bidchol, Luitgard Graul-Neumann, Ashish Gupta, Ute Hehr, Davor Lessel, Sean Nader, Hitesh Shah, Julia Wickert, Kerstin Kutsche

**Affiliations:** Department of Medical Genetics, Kasturba Medical College, Manipal University, Manipal, Karnataka India; Ambulantes Gesundheitszentrum der Charité, Campus Virchow, Humangenetik, Universitätsmedizin Berlin, Berlin, Germany; Center for and Department of Human Genetics, University of Regensburg, Regensburg, Germany; Institute of Human Genetics, University Medical Center Hamburg-Eppendorf, Martinistraße 52, 20246 Hamburg, Germany; Kinderorthopädie, Schön Klinik Vogtareuth, Prien am Chiemsee, Germany; Pediatric Orthopedic Services, Department of Orthopedics, Kasturba Medical College, Manipal University, Manipal, Karnataka India

**Keywords:** Larsen syndrome, *FLNB*, Mutation, Gain-of-function, Autosomal-dominant, Pre-mRNA splicing

## Abstract

**Background:**

Larsen syndrome is an autosomal dominant skeletal dysplasia characterized by large joint dislocations and craniofacial dysmorphism. It is caused by missense or small *in-frame* deletions in the *FLNB* gene. To further characterize the phenotype and the mutation spectrum of this condition, we investigated seven probands, five sporadic individuals and a mother-son-duo with Larsen syndrome.

**Methods:**

The seven patients from six unrelated families were clinically and radiologically evaluated. All patients were screened for mutations in selected exons and exon-intron boundaries of the *FLNB* gene by Sanger sequencing. *FLNB* transcript analysis was carried out in one patient to analyse the effect of the sequence variant on pre-mRNA splicing.

**Results:**

All patients exhibited typical facial features and joint dislocations. Contrary to the widely described advanced carpal ossification, we noted delay in two patients. We identified the five novel mutations c.4927G > A/p.(Gly1643Ser), c.4876G > T / p.(Gly1626Trp), c.4664G > A / p.(Gly1555Asp), c.2055G > C / p.Gln685delins10 and c.5021C > T / p.(Ala1674Val) as well as a frequently observed mutation in Larsen syndrome [c.5164G > A/p.(Gly1722Ser)] in the hotspot regions. *FLNB* transcript analysis of the c.2055G > C variant revealed insertion of 27 bp intronic sequence between exon 13 and 14 which gives rise to *in-frame* deletion of glutamine 685 and insertion of ten novel amino acid residues (p.Gln685delins10).

**Conclusions:**

All seven individuals with Larsen syndrome had a uniform clinical phenotype except for delayed carpal ossification in two of them. Our study reveals five novel *FLNB* mutations and confirms immunoglobulin-like (Ig) repeats 14 and 15 as major hotspot regions. The p.Gln685delins10 mutation is the first Larsen syndrome-associated alteration located in Ig repeat 5. All mutations reported so far leave the filamin B protein intact in accordance with a gain-of-function effect. Our findings underscore the characteristic clinical picture of *FLNB*-associated Larsen syndrome and add Ig repeat 5 to the filamin B domains affected by the clustered mutations.

## Background

Mutations in the *FLNB* (filamin B; MIM 603381) gene cause a group of skeletal dysplasias comprising spondylocarpotarsal syndrome (SCT, MIM 272460), Larsen syndrome (LS, MIM 150250), atelosteogenesis I and III (AOI, MIM 108720; AOIII, MIM 108721), and boomerang dysplasia (BD, MIM 112310) [1]. Homozygous and compound heterozygous *FLNB* null alleles cause SCT which is characterized by fusions of vertebral bodies, carpal and tarsal bones [2]. AOI and AOIII are dominant lethal skeletal dysplasias with vertebral abnormalities, joint dislocations, poorly modeled long bones, absent or hypoplastic bones, and disharmonious skeletal maturation [3, 4]. BD is a severe skeletal phenotype that is lethal *in utero*. Radiological findings of BD include irregular ossification of limb bones and vertebrae, under development of the acetabulum, and hypoplastic, boomerang shaped femora from which the disorder derives its name [5, 6].

LS, which was first described in 1950, has a wide range of clinical and radiological features involving joint dislocations of the hip, knee and elbow, equinovarus foot deformities, early and extra ossification of carpal bones, and characteristic facial features [7]. Facial features include depressed nasal bridge, prominent forehead, and telecanthus [8]. Cleft palate, scoliosis, cervical kyphosis, and short stature are seen in affected individuals [9–11]. Hearing loss occurs due to malformation of auditory ossicles [12–14]. In 2004, heterozygous missense mutations in *FLNB* have been reported in four unrelated individuals and a mother-child duo with LS [1]. Since then, very few reports have been published on mutations causing LS [1, 15, 16]. An autosomal recessive inheritance pattern has been reported in several families originating from La Reunion Island [17]. Recently, a homozygous missense mutation in the *B4GALT7* gene (MIM 604327) has been identified in individuals with this form of LS [18].

Filamin B belongs to the filamin family consisting of the three homologous proteins FLNA (MIM 300017), FLNB, and FLNC (MIM 102565). Filamins are large actin-binding cytoplasmic proteins that help reorganizing the actin cytoskeleton, stabilize cortical F-actin networks, and link them to cellular membranes by binding to transmembrane receptors or ion channels [19, 20]. Filamin B is expressed in various tissues and is predominantly present in developing mouse vertebral bodies and human fetal epiphyseal growth plate chondrocytes [1]. The phenotype of *Flnb* knockout mice corresponds well to the clinical features of SCT [2, 21–23].

Filamins contain an F-actin binding domain (ABD) at the N-terminus and a rod segment consisting of up to 24 immunoglobulin-like folds (Ig repeats). Two hinge regions separate the 24 Ig repeats into two rod domains, and at the C-terminus a filamin repeat mediates dimerization. The ABD is composed of two calponin homology (CH) domains (CH1 and CH2) separated by a linker [20]. Mutations in *FLNB* cluster in specific protein domains. Mutations associated with AOI and AOIII are present in the CH2 domain and Ig repeats 2, 5, 6, 14, and 15 [1, 4, 24], while amino acid substitutions causing BD are exclusively located in the CH2 domain [6, 24]. The missense mutations and small *in-frame* deletions found in LS have been detected both in the CH2 domain and the Ig repeats 2, 13, 14, 15, 17, and 23 [1, 15, 16, 24–26]. Dominant *FLNB* mutations have been proposed to result in increased F-actin binding affinity of filamin B or dysregulation of the hinge region [24, 27].

Here we describe clinical and radiological features of seven individuals with LS and report novel causative *FLNB* mutations, including the first *in-frame* deletion-insertion in the filamin B repeat 5.

## Methods

### Clinical investigation

We recruited seven patients from six unrelated families after detailed clinical and radiological evaluation. Clinical and molecular findings in patients 1–7 are summarized in Table 1. Informed consents for participation, publication of photographs, and blood sample collection were obtained from the patients’ and/or the patients’ parents/legal guardians, according to the Declaration of Helsinki and the national legal regulations (e.g. German Genetic Diagnosis Act [GenDG]).

**Table 1:** Clinical, radiological and molecular findings in seven patients with Larsen syndrome

Clinical features	Patient 1	Patient 2	Patient 3	Patient 4	Patient 5	Patient 6	Patient 7
Gender	Male	Male	Female	Male	Male	Female	Male
Ethnicity	Indian	Indian	Indian	Indian	German	German	German
Age at last follow-up	10 y	3 y	9 y	6 y	42 y	39 y	4 y
Prenatal growth deficiency	–	+	–	+	–	–	–
Birth weight	3.25 kg	2.5 kg	3.25 kg	2.2 kg	3.58 kg	ND	3.88 kg
Birth height	ND	ND	ND	ND	52 cm	ND	51 cm
OFC at birth	ND	ND	ND	ND	37 cm	ND	37 cm
Clinical phenotype							
Intellectual disability	–	–	–	–	–	–	–
Prominent forehead	–	+	+	+	+	+	+
Midface hypoplasia	+	–	–	+	+	+	+
Hearing loss	–	–	–	–	–	–	–
Telecanthus	+	+	+	+	+	+	+
Depressed nasal bridge	+	+	+	+	+	+	+
Cleft palate	+	+	–	–	–	–	–
Teeth anomaly	–	–	–	–	–	–	–
Broad fingertips	+	–	+	+	+	+	+
Short nails	–	–	–	–	–	–	–
Airway abnormality	–	–	–	–	–	–	–
Cardiac defect	–	–	–	–	–	–	–
Cryptorchidism	–	–	NA	–	–	NA	–
Spinal cord compression	–	–	–	–	–	–	–
Skeletal phenotype							
Short stature	–	–	–	–	+	+	–
Joint laxity	+	+	+	+	+	+	+
Cylindrical fingers	–	+	+	+	+	+	+
Pectus deformity	–	–	–	–	–	–	–
Scoliosis	–	+	–	–	cervical scoliosis	+	–
Abnormal thumbs/halluces (broad/bifid/spatulate)	+	+	+	–	+	+	+
Club foot	+	+	+	+	+	+	+
Hip dislocation	–	–	+	–	+	+	+
Knee dislocation	+	+	+	+	+	+	+
Elbow dislocation	+	+	–	+	+	+	+
Radiographic findings							
Cervical vertebral dislocation	–	+	+	–	cervical instability	–	+
Cervical scoliosis/kyphosis	+	+	+	–	+	–	+
Wedge/block vertebrae	–	–	–	–	–	–	+
Supernumerary carpal bones	+	–	+	–	+	n/a	n/a
Delayed bone age	–	+	–	+	n/a	n/a	–
Supernumerary tarsal bones	–	–	+	+	n/a	n/a	n/a
Molecular findings							
*FLNB* sequence change (according to mRNA RefSeq NM_001164317.1 from GenBank)	c.4927G > A	c.5164G > A	c.4876G > T	c.4664G > A	c.2055G > C r.2055delgins28	c.5021C > T	c.5021C > T
*de novo*	+	+	absent in mother	+	+	n/a	inherited
Amino acid change (according to protein RefSeq NP_001157789.1 from GenBank)	p.(Gly1643Ser)	p.(Gly1722Ser)	p.(Gly1626Trp)	p.(Gly1555Asp)	p.Gln685delins10	p.(Ala1674Val)	p.(Ala1674Val)
Amino acid change (according to protein RefSeq NP_001448.2 from GenBank) [24]	p.(Gly1612Ser)	p.(Gly1691Ser)	p.(Gly1595Trp)	p.(Gly1524Asp)	p.Gln685delins10	p.(Ala1643Val)	p.(Ala1643Val)
Protein domain	Ig repeat 15	Ig repeat 15	Ig repeat 15	Ig repeat 14	Ig repeat 5	Ig repeat 15	Ig repeat 15
Novel/reported	Novel	Reported [15]	Novel	Novel	Novel	Novel	Novel

Legends: *+* present, − absent, *Ig* immunoglobulin-like, *NA* Not applicable, *n/a* not analysed, *ND* no data, *y* years. Description of the mutations on protein level is given between brackets when RNA nor protein has been analysed. An “r.” is used to indicate that the change is described at RNA level

### Sequencing of *FLNB*

DNA was isolated from leukocytes by standard procedures. We amplified selected exons and exon-intron boundaries of the *FLNB* gene (exons 11–20 and 22–35) [GenBank:NM_001164317.1] from genomic DNA. We used the mRNA RefSeq NM_001164317.1 as reference sequence and not NM_001457.3 [24] as the aforementioned RefSeq represents the longest *FLNB* transcript and encodes the longest protein. Primer sequences are available on request. Amplicons were directly sequenced using the ABI BigDye Terminator Sequencing Kit (Applied Biosystems, Darmstadt, Germany) and an automated capillary sequencer (ABI 3500; Applied Biosystems). Sequence electropherograms were analysed using the Sequence Pilot software (JSI Medical Systems, Kippenheim, Germany). Genotyping was carried out with the AmpFLSTR SGM plus PCR Amplification Kit (Applied Biosystems) to confirm paternity and maternity.

### *FLNB* transcript analysis

RT-PCR sequencing was performed as previously described [28]. Briefly, we isolated RNA using the PAXgene Blood RNA Kit IVD (Qiagen, Hilden, Germany) and performed RT-PCR using the OneStep RT-PCR Kit (Qiagen). Primer sequences were as follows: Forward primer: 5′-CCTGGCGAATATGCTGTTCACATC-3′ and reverse primer: 5′-CGGGGTGTATGAGCATGCATATGT-3′. Amplicons were extracted from the agarose gel using the QIAquick Gel Extraction Kit (Qiagen) and were subsequently sequenced. Sequence traces were assembled, aligned and analysed with the Seqman software (DNASTAR Lasergene, Madison, WI, USA).

## Results

### Clinical evaluation

**Patient 1** is a 10-year-old boy. He was born to non-consanguineous parents at term by cesarean section. Birth weight was 3.25 kg (normal). He cried immediately after birth. He was referred for evaluation with bilateral club feet and cleft palate noted at birth and hyper-extensibility of both knee joints noted since 4 months of age. He was operated for cleft palate at 15 months of age. He has undergone multiple corrective orthopedic surgeries for clubfeet. He had recurrent lower respiratory tract infections until 2 years of age and he was found to have significant gastroesophageal reflux and was medically treated. After 2 years of age, he had no history suggestive of breathing difficulty or noisy breathing. He achieved head holding at 3 months, sitting at 8 months, walked with support at 15 months and walked without support at 2 years. He started smiling at 4 months, babbling at 5 months, spoke monosyllables at 1 year and bisyllables at 2 years.

**Fig. 1:**
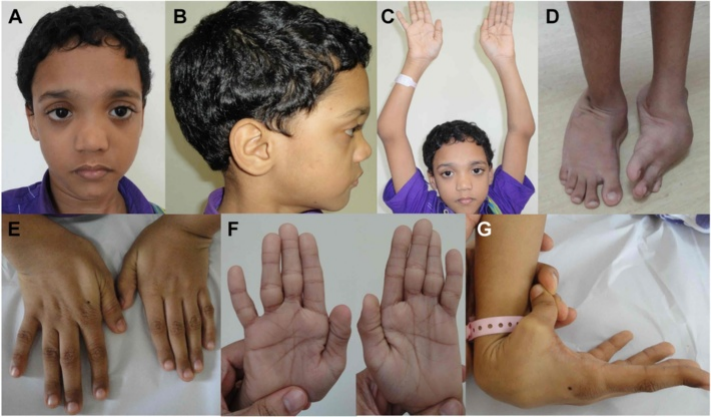
Photographs of patient 1 at the age of 10 years. He presented with flat face, depressed nasal bridge, telecanthus, long philtrum (**a** and **b**), bilateral dislocated elbows (**c**), bilateral club feet (**d**), broad fingertips, spatulate thumbs, abnormal palmar creases (**e** and **f**), and joint laxity (**g**)

At 10 years, his height was 132 cm, OFC 52.5 cm and weight 29.3 kg (all are normal for age). He had facial dysmorphism with depressed nasal bridge, mid face hypoplasia, telecanthus and long philtrum (Fig. 1a and b). Bilateral subluxation of elbow joints and knees and bilateral club feet were present (Fig. 1c and d). He had bilateral 5^th^ finger clinodactyly, spatulate thumbs, broad fingertips, abnormal palmar creases and joint laxity (Fig. 1e, f and g). Hip and wrist joints were normal. Skin, hair, nails and teeth were normal. There was no evidence of hearing defect or compressive myelopathy. Other examinations were unremarkable, such as echocardiography and ophthalmology. At present, he is class four student and is average in studies.

**Fig. 2:**
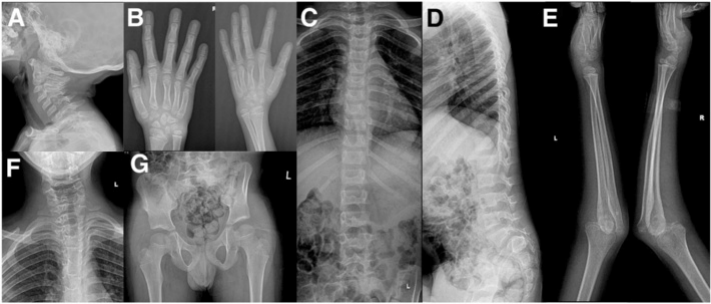
Radiographs of patient 1 at the age of 8 years. He showed normal cervical spine without instability (**a**), supernumerary carpal bones (**b**), normal thoracolumbar spine (**c** and **d**), bilateral elbow dislocation (**e**), cervical-dorsal scoliosis (**f**), and normal hip joints (**g**)

Skull radiography revealed normal cervical spine (Fig. 2a). Radiographs were suggestive of supernumerary carpal bones, short metacarpals (Fig. 2b), normal thoracolumbar spine (Fig. 2c and d), bilateral elbow joints dislocation (Fig. 2e) and cervical-dorsal scoliosis (Fig. 2f). Radiograph of pelvis with both hips was unremarkable (Fig. 2g). His complete blood count, serum calcium, serum phosphorous, alkaline phosphatase levels and complete urine analysis were unremarkable.

**Patient 2** is a 3-year-old boy, born to second degree consanguineous parents at term by cesarean section, in view of prolonged labor. Birth weight was 2.5 kg (−2 SD). He cried immediately after birth. He is the only child of his parents. Maternal antenatal scan at 6 months of gestation was suggestive of fetal limb deformity that was confirmed after birth. He was referred for evaluation of deformity of bilateral feet, elbows and right knee noted since birth. At birth, he was also found to have cleft palate for which he was operated at 1 year of age.

**Fig. 3:**
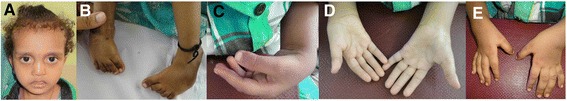
Photographs of patient 2 at the age of 3 years. He had prominent forehead, telecanthus (**a**), bilateral club feet (**b**), spatulate thumb (**c**), and cylindrical fingers (**d** and **e**)

At 6 weeks, his height was 52 cm (−2 SD), OFC 37 cm (−2 SD) and weight 3.3 kg (−2 SD). He had prominent forehead, depressed nasal bridge, long philtrum, telecanthus (Fig. 3a), and high arched palate. His hair was curly (Fig. 3a), teeth and nails were normal. Hypermobility of bilateral hip joints was noted along with bilateral dislocated knees, elbows and bilateral clubfeet (Fig. 3b). He had spatulate thumbs (Fig. 3c), cylindrical fingers (Fig. 3d and e) with short and broad fingertips, and joint laxity. Hearing was normal. There was no evidence of compressive myelopathy. All other examinations were unremarkable. Echocardiography showed normal heart. He achieved head control at 5 months, roll over at 7 months, sitting with support at 10 months, sitting without support at 15 months, crawling at 20 months. He started babbling at 6 months, spoke bisyllables at 15 months and few words with meaning at 20 months.

**Fig. 4:**
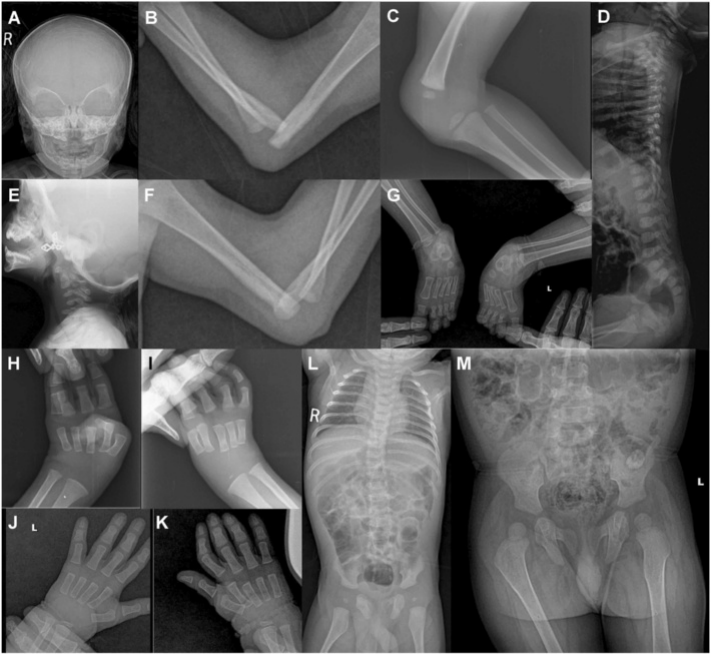
Radiographs of patient 2. He had normal skull (**a**), bilateral elbow dislocation (**b** and **f**), absent patella and dislocated knee (**c**), coronal clefts in vertebrae (**d**), cervical kyphosis with subluxation at C4-C5 (**e**), double calcaneum ossification with club feet (**g**), delayed carpal ossification at 3 months (**h** and **i**), and 1 year (**j** and **k**), thoracolumbar scoliosis and small capital femoral epiphysis at 3 months (**l**), and at 1 year (**m**)

Radiograph of skull was unremarkable (Fig. 4a). Ultrasound of knees at 6 weeks of age suggested absent right patella. Radiographs showed bilateral dislocated elbows (Fig. 4b and f), absent right patella (Fig. 4c), coronal clefts in vertebrae (Fig. 4d), cervical vertebral kyphosis with subluxation at C4-C5 (Fig. 4e), and double calcaneum ossification with club feet (Fig. 4g). Radiographs at 3 months (Fig. 4h and i) and 1 year (Fig. 4j and k) suggested delayed carpal ossification. Radiographs at 3 months and 1 year showed thoracolumbar scoliosis (Fig. 4l) and small capital femoral epiphysis (Fig. 4l and m). Thyroid profile was done in view of delayed bone age and was found to be normal. His complete blood counts, serum calcium, serum phosphorous, alkaline phosphatase, and complete urine analysis were normal.

**Patient 3** is a 9-year-old female, born to third degree consanguineous parents at term by cesarean section for breech presentation. Birth weight was 3.25 kg (normal). She cried immediately after birth. She was referred for evaluation in view of bilateral dislocation of hip and knee joints and bilateral clubfeet. She has undergone multiple corrective orthopedic surgeries for club feet and hip dislocation. She started walking at 1 year and spoke simple sentences at 2 years.

**Fig. 5:**
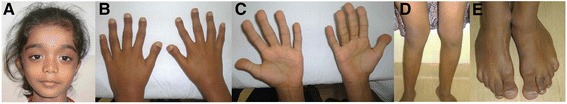
Photographs of patient 3 at the age of 9 years. She presented with prominent forehead, depressed nasal bridge, telecanthus (**a**), broad fingertips, spatulate thumbs (**b** and **c**), bilateral knee dislocation (**d**), broad great toes, and bilateral club feet (**e**)

At 2 years and 2 months, her height was 90 cm, OFC 48.5 cm and weight 11 kg (all are normal for age). She had prominent forehead, depressed nasal bridge, and telecanthus (Fig. 5a). She had spatulate thumbs, broad fingertips (Fig. 5b and c), and joint laxity. Joint laxity was noted in both knee joints (Fig. 5d), bilateral club feet and broad great toes were also noted (Fig. 5e). Bilateral elbow and wrist joints were normal. Skin, hair, and nails were normal. At 9 years of age, there was no evidence of hearing defect or compressive myelopathy. She is presently in 3^rd^ grade and is good at studies.

**Fig. 6:**
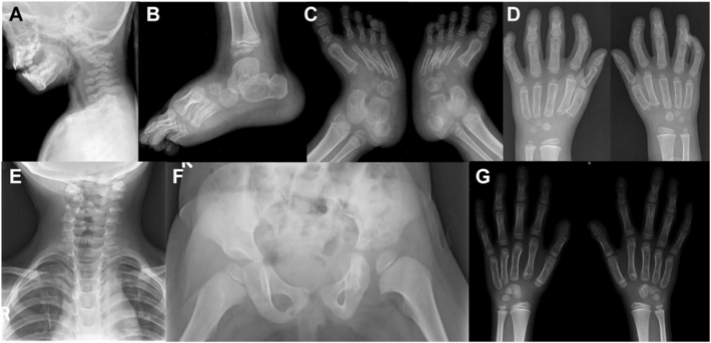
Radiographs of patient 3. She had cervical kyphosis with subluxation at C3-C4 (**a**), accessory calcaneal epiphysis (**b**), club feet with metatarsus adductus (**c**), advanced carpal ossification at 22 months (**d**), failure of fusion of posterior element of cervical spine with mild scoliosis (**e**), normal right hip joint with increased medial joint space on left hip joint (**f**), and normal bone at 4 years 7 months (**g**)

Radiographs revealed cervical kyphosis, subluxation at C3-C4 (Fig. 6a), and failure of fusion of posterior elements of cervical spine with mild scoliosis (Fig. 6e). Accessory calcaneal ossification (Fig. 6b) and clubfeet with metatarsus adductus (Fig. 6c) were also noted. Advanced carpal ossification was seen in radiographs at 22 months (Fig. 6d) of age though carpal ossification was age appropriate at 4 years and 7 months (Fig. 6g). She had normal right hip joint but increased medial joint space on left hip joint (Fig. 6f). Echocardiography was normal. Her complete blood counts, serum calcium, serum phosphorous, alkaline phosphatase, thyroid profile, and complete urine analysis were unremarkable.

**Patient 4** is a 6-year-old boy, born to non-consanguineous parents at term by vaginal delivery with birth weight of 2.2 kg (−2 to −3 SD). He cried immediately after birth. He had one healthy elder brother. He was referred for evaluation with bilateral clubfeet, bilateral elbow and knee dislocations noted at birth. He achieved head holding at 5 months, roll over at 8 months, sitting at 9 months, standing at 13 months, walking with support at 15 months, and walking without support at 2 years.

**Fig. 7:**
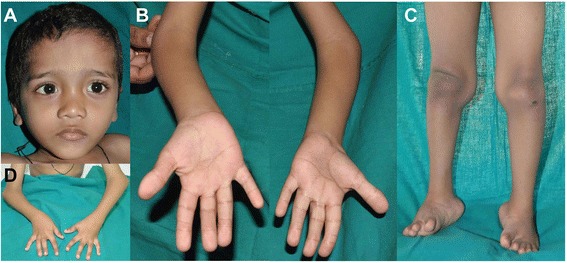
Photographs of patient 4 at the age of 6 years. He showed prominent forehead, depressed nasal bridge, telecanthus (**a**), bilateral elbow dislocation, broad fingertips (**b** and **d**), bilateral knee dislocation, and bilateral club feet (**c**)

At 6 years, his height was 110 cm and weight 22 kg (both normal for age). He had prominent forehead, mid face hypoplasia, telecanthus, downslanting palpebral fissures, and long philtrum (Fig. 7a). He had long cylindrical fingers with broad fingertips (Fig. 7b and d), and mild joint laxity. He also had bilateral elbow and knee dislocation and clubfeet (Fig. 7b, c and d). He had flexion deformity at bilateral elbow with pterygium over cubital fossa (Fig. 7b). Bilateral hip and wrist joints were normal. Skin, hair, and nails were normal.

**Fig. 8:**
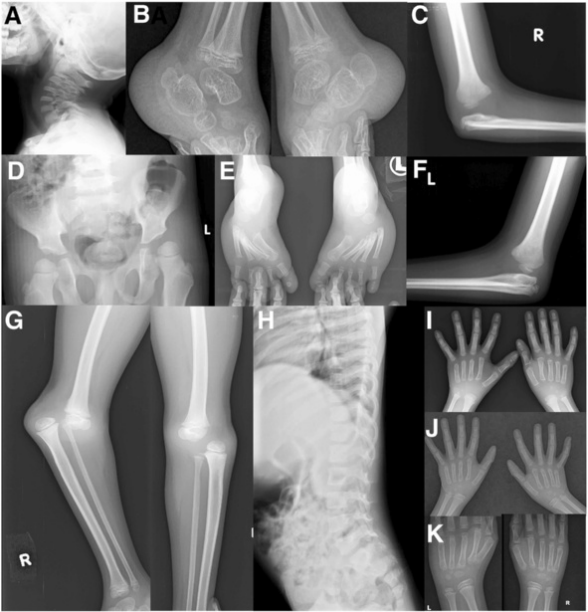
Radiographs of patient 4. He had normal cervical spine (**a**), double ossification of calcaneum in both feet (**b**), bilateral elbow dislocations (**c** and **f**), normal hip joint (**d**), bilateral metatarsus adductus (**e**), bilateral knee dislocations (**g**), normal thoracolumber spine (**h**), delayed carpal ossification at 4 years (**i**), 5 years (**j**), and 6 years (**k**) of age as well as abnormal distal phalanx of thumbs and small distal phalanges (**j**)

He had normal cervical spine (Fig. 8a). Bilateral double ossification of calcaneum (Fig. 8b), bilateral elbow (Fig. 8c and 8f) and knee dislocations (Fig. 8g), and bilateral metatarsus adductus (Fig. 8e) were noted. Radiographs of hip and thoracolumbar spine were unremarkable (Fig. 8d and h). Radiographs at 4 years, 5 years and 6 years of age revealed delayed carpal ossification (Fig. 8i, j and k) and showed abnormal distal phalanx of thumbs and small distal phalanges (Fig. 8j). Echocardiography was normal. His complete blood count, serum calcium, serum phosphorous, alkaline phosphatase, thyroid profile, and complete urine analysis were unremarkable.

**Fig. 9:**
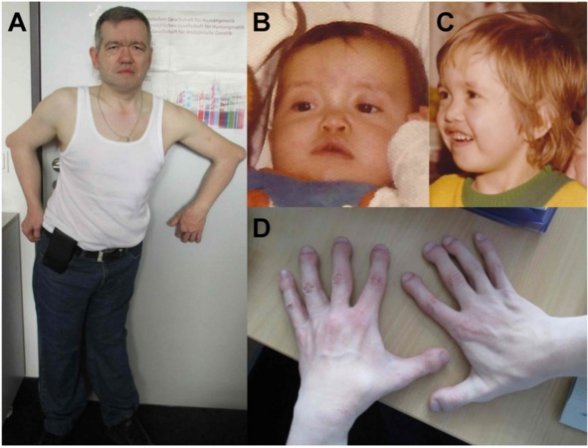
Photographs of patient 5. He is a 42-year-old male with multiple joint dislocations of the hips, knees and elbows and secondary deformities (**a**). Patient 5 at age 6 months (**b**), and 4 years (**c**). He showed craniofacial dysmorphism with prominent forehead, low nasal bridge, telecanthus and flattened face (**a**-**c**), cylindrical fingers and spatulate thumbs (**d**)

**Patient 5** is a 42-year-old man, the second son of unaffected and non-consanguineous parents. He was born, followed by unremarkable pregnancy, at 40 weeks of gestation with a birth weight of 3,580 g (+0.37 SD), length of 52 cm (+0.19 SD) and OFC of 37 cm (+1.92 SD). At birth, he presented with dislocation of knee joints, anterior dislocation of the tibia, bilateral pronounced club feet, and distinct dislocation of both hips. Facial dysmorphism with a flat face and nose, prominent forehead, telecanthus, and myopia was noted (Fig. 9a, b and c). He had broad distal phalanges of the hands (Fig. 9d), supernumerary carpal bones, and 13 pairs of ribs. During the neonatal period, knee dislocation was treated with manipulation and cast application. Later, club feet were treated with metatarsal osteotomy with posterior soft tissue release. The correction of feet was maintained with feet orthosis until 14 years of age. An arthrodesis of the right knee joint was performed for chronic pain. He later developed a cervical scoliosis with occipito-craniocervical instability. Cervical spine instability was treated with occipitocervical fusion and a return displacement of the soft palate was conducted. No abnormalities of the cardiovascular system were observed. At the age of 37 years, he developed chronic myeloid leukemia. The patient holds a degree in natural science. He is severely limited in his mobility due to the skeletal abnormalities.

**Patient 6** is a 39-year-old woman, born to non-consanguineous parents. Normal vaginal delivery took place at full term. Birth measurements were in the normal range. Her parents, sister and brother were unaffected, similar to her first son. Her second son was affected (see patient 7). Congenital club feet were treated with tenotomy of Achilles tendon. Bilateral hip dislocation was treated with several orthopedic surgeries. In early childhood, she could walk with braces. She still had club feet deformity. At the age of 29, she became wheelchair-dependent due to hip dislocation and club feet.

**Fig. 10:**
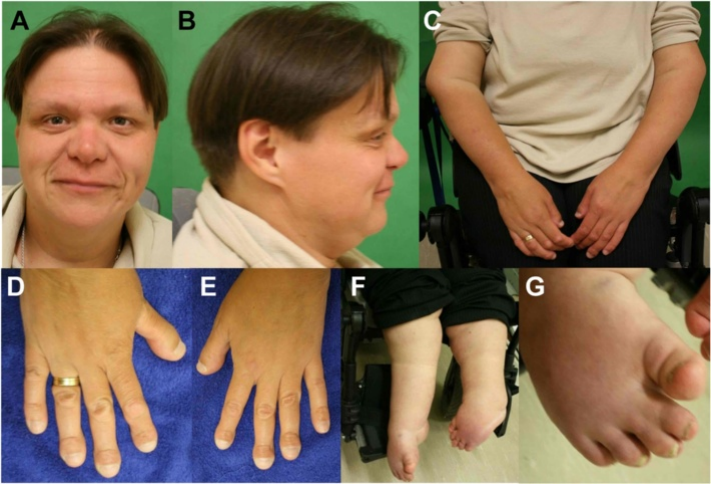
Photographs of patient 6. She showed prominent forehead, depressed nasal bridge, long philtrum, telecanthus (**a** and **b**), bilateral dislocated elbows (**c**), cylindrical fingers with short and broad fingertips, spatulate thumbs (**d** and **e**), bilateral fixed club feet deformities (**f**) with partial syndactyly of toes 2/3 on the right (**g**)

**Fig. 11:**
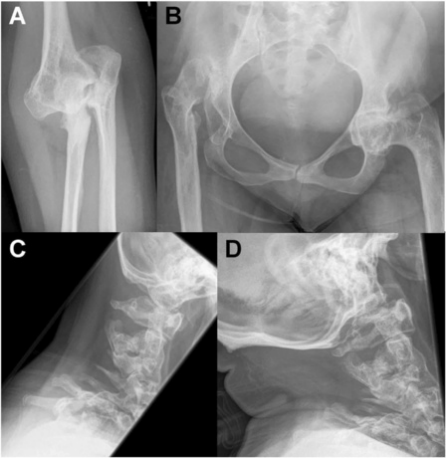
Radiographs of patient 6. Dislocation of the right elbow: olecranon dislocated to the ulnar side without articulation (**a**). Right side of the hip AP: cranial dislocated and ossified with neojoint X; left side: varus position with arthrotic joint, sclerotic fringe and small intra-articular space and spinoiliacal osteophyte (**b**). Functional graphs of the cervical spine did not provide evidence for cervical instability but showed deformed cervical vertebral bodies with fusion C3/C4(**c** and **d**). AP: anteroposterior

At the age of 39 years, physical examination revealed height of 145 cm (−3.5 SD), OFC of 57 cm (−1.8 SD), and weight of 85 kg (+1.8 SD). She had prominent forehead, depressed nasal bridge, long philtrum, and telecanthus (Fig. 10a and b). She had bilateral dislocated elbows (Fig. 10c) and cylindrical fingers with short and broad fingertips and spatulate thumbs (Fig. 10d and e). She had bilateral fixed clubfeet deformities with partial syndactyly of toes 2/3 on the right (Fig. 10f and g). Wrist joints were normal and there was no pectus deformity. Teeth and nails were normal. Hearing was normal. Numbness of the upper extremities was suggestive of compressive myelopathy. Radiographs confirmed bilateral dislocated elbows (Fig. 11a), hip dislocation with ossified right hip joint (Fig. 11b), and deformities of cervical vertebral bodies (Fig. 11c and d).

**Patient 7** is the son of patient 6. During pregnancy bilateral foot deviation was detected by ultrasound. He was born to non-consanguineous parents by normal vaginal delivery at term. Birth weight was 3.88 kg (+0.5 SD), length 51 cm (−1.2 SD) and OFC 37 cm (+1.6 SD). Apgar scores were 10/10. Congenital bilateral club feet were treated with serial castings and tenotomy of Achilles tendon. He also had dislocations of both knees. At 14 months, he could sit. He walked without support at the age of 2 years with above-knee braces.

**Fig. 12:**
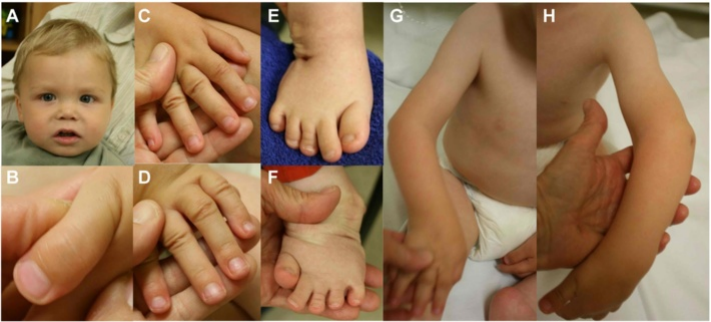
Photographs of patient 7 at one and a half years. He showed prominent forehead, depressed nasal bridge, telecanthus (**a**), spatulate thumbs (**b**), hands with broad fingertips (**c** and **d**), feet with broad toes (**e** and **f**), and bilateral elbow dislocation (**g** and **h**)

**Fig. 13:**
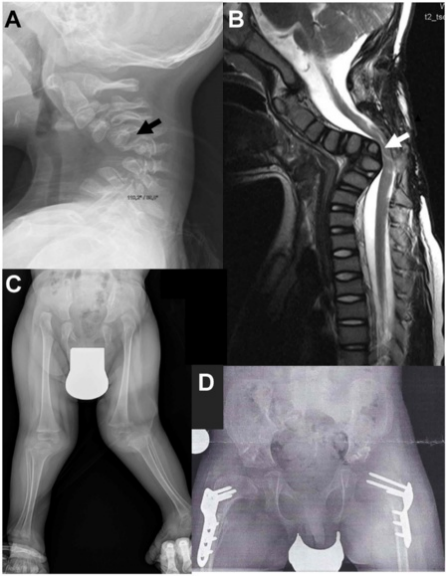
Radiographs of patient 7. Cervical lateral spine at 2 4/12 years: Cervical kyphosis of ~70° with dorsal cervical listhesis of vertebral bodies C4/C5 and C5/C6 (black arrow), dysplasia of vertebral body C6 (**a**). Cervical myelogram at 3 years: cord compression and myelopathy at C5/C6 (white arrow) (**b**). Radiograph of the hip and legs AP: note bilateral hip dysplasia with flat acetabulum, valgus position of femoral necks, luxation of both hip joints with cranial position of the femoral head and knee luxation on both sides (**c**). Radiograph of the hip AP: corrected position after operative hip replacement by acetabular reconstruction and femoral osteotomy (**d**). AP: anteroposterior

At one and a half years, he was presented to the genetic department. His height was 79 cm (−1.6 SD), OFC 47 cm (−0.9 SD) and weight 10 kg (−1.3 SD). He showed flat facial appearance, depressed nasal bridge, mid face hypoplasia, telecanthus, and long philtrum (Fig. 12a). He had bilateral spatulate thumbs (Fig. 12b), cylindrical shaped fingers (Fig. 12c and d), feet with broad toes (Fig. 12e and f), and joint laxity. Bilateral subluxation of elbow joints with fixed flexion contractures was apparent (Fig. 12g and h). Wrist joints were normal. No deformity of the sternum was noted. Skin, hair, nails, and teeth were normal. Ultrasound of heart and abdomen was normal. There was no evidence of a hearing defect. External genitalia were normal. Cervical kyphosis of about 70° was apparent at the age of 2 years and 4 months (Fig. 13a). At 2 years, his knee joints were unstable and treated with above-knee braces. At 3 years, the cervical kyphosis had progressed to 150° (Fig. 13b). At this age, he had frequent respiratory infections and breathing problems due to compressive myelopathy verified by magnetic resonance imaging (Fig. 13b). Laryngoscopy revealed tracheal stenosis and laryngomalacia. A posterior spondylodesis from the third to the sixth cervical vertebral body (with bone implantation of the iliac crest) was performed at the age of 3 years and later on stabilized with titanium plates by four orthopedic procedures. He underwent open reduction of bilateral hip dislocation at 4 years of age (Fig. 13c and d). His intellectual development was normal.

### *FLNB* mutations

Sanger-sequencing of exons 11–20 and 22–35 of the *FLNB* gene was performed in five unrelated patients and a mother-son-duo, all with a clinical diagnosis of LS. Patient 1 was found to be heterozygous for the mutation c.4927G > A [p.(Gly1643Ser)] in exon 29, patient 2 for c.5164G > A [p.(Gly1722Ser)] in exon 30, patient 3 for c.4876G > T [p.(Gly1626Trp)] in exon 29, patient 4 for c.4664G > A [p.(Gly1555Asp)] in exon 28, and patient 5 for c.2055G > C predicting the amino acid change p.(Gln685His) in exon 13 (Table 1). The c.5021C > T [p.(Ala1674Val)] mutation in exon 30 was found in both the affected mother (patient 6) and her affected son (patient 7) (Table 1). None of the *FLNB* variants was annotated in the 1000 Genomes Project, Exome Variant Server and The Exome Aggregation Consortium databases. All non-synonymous variants are predicted to have damaging impact on protein function by the Combined Annotation Dependent Depletion (CADD) scoring system [29] (data not shown). Except of the missense change p.(Gly1722Ser), which is the most common *FLNB* mutation found in LS-affected subjects (corresponding to p.(Gly1691Ser) according to RNA RefSeq NM_001457.3) [24], all other mutations have not yet been described in LS. The healthy parents of patients 1, 2, 4, and 5 did not carry the *FLNB* mutation indicating *de novo* occurrence in the affected individuals (Table 1; paternity confirmed). The father of patient 3 and the parents of patient 6 were not available for testing.

### *FLNB* transcript analysis

**Fig. 14:**
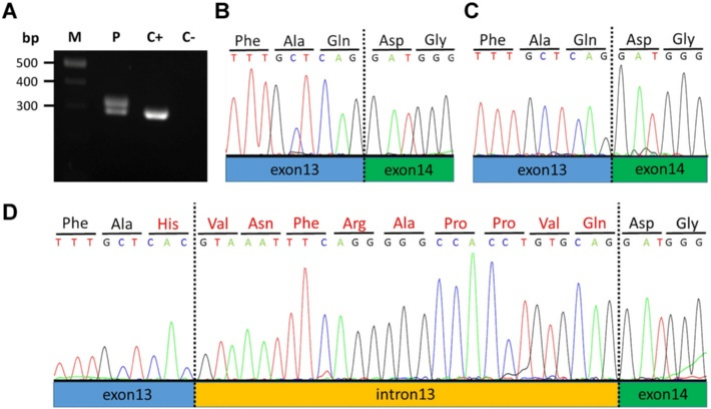
*FLNB* transcript analysis in patient 5 with the heterozygous mutation c.2055G > C. **a** *FLNB* transcript analysis was performed using lymphocyte-derived RNA of the patient (P) and a healthy individual (C+). The 294-bp wild-type RT-PCR amplicon was amplified in both the patient and the control (C+), while no product was yielded in the negative control (C-, no template). A second, larger PCR product of ~330 bp was only obtained in the patient (P). The 500, 400 and 300 bp reference bands of the molecular marker (M) are indicated. **b** Sequence trace of the exon 13 (blue box)-exon 14 (green box) junction in a healthy individual. **c** Sequence trace of the smaller PCR product (294 bp) of patient 5 revealed wild-type sequence at the exon 13-exon 14 junction. Note the guanine as the last nucleotide in exon 13. **d** Sequence trace of the larger RT-PCR amplicon (321 bp) of the patient showed the mutant base cytosine at the last position of exon 13 (changing the codon for glutamine to histidine [indicated in red]) and an *in-frame* inclusion of the first 27 bp of intron 13 (orange box; codons for nine FLNB-unrelated amino acid residues are highlighted in red). Codons are underlined and amino acid residues are indicated in the three-letter code above each electropherogram

The heterozygous c.2055G > C transversion in patient 5 is predicted to result in substitution of glutamine at position 685 by histidine. However, the mutation is located at the last nucleotide of exon 13 and could result in altered pre-mRNA splicing. We investigated the effect of this sequence change on pre-mRNA splicing. RT-PCR on RNA isolated from lymphocytes of patient 5 yielded two amplicons, one amplicon of the expected wild-type size (294 bp) and one larger amplicon of ~330 bp that was not obtained in a healthy control individual (Fig. 14a). Sequence analysis of the smaller amplicon revealed the wild-type base guanine at the exon 13-exon 14 junction in the 294-bp fragment of both a control individual (Fig. 14b) and patient 5 (Fig. 14c). In contrast, in the larger amplicon the mutated base cytosine was present at the last position of exon 13 and the first 27 bp of intron 13 were inserted between exons 13 and 14 (Fig. 14d). Thus, this aberrant transcript variant is produced from the c.2055C-mutant allele and contains ten *FLNB*-unrelated codons, starting with the codon for histidine (last codon of exon 13) followed by nine codons from intron 13 sequence (Fig. 14d). Therefore, the correct description of this *FLNB* mutation in patient 5 is c.2055G > C on DNA level, r.2055delgins28 on RNA level and p.Gln685delinsHisValAsnPheArgAlaProProValGln/p.Gln685delins10 on protein level after RNA analysis (Table 1).

## Discussion

We describe here the clinical phenotype of four Indian and three German individuals affected by LS. Two Indian families reported consanguinity which was, however, not validated experimentally. In each of the patients, a causative mutation in *FLNB* was identified. The reported individuals appear to have similar clinical features, which in general correspond well to what has been described in LS before. They had multiple joint dislocations at birth, with hip (4/7), knees (7/7), and elbows (6/7) most frequently affected. Other common features were prominent forehead (6/7), mid face hypoplasia (5/7), telecanthus (7/7), depressed nasal bridge (7/7), abnormal thumbs (6/7), broad fingertips (6/7), cylindrical fingers (6/7), and club feet (7/7). Less frequently, cleft palate (2/7) and recurrent respiratory tract infections were found. One of the seven subjects (patient 7) had compressive myelopathy as a serious neurological complication.

Radiologically, cervical vertebral abnormalities (4/7) (dislocation or instability), kyphoscoliosis (5/7), supernumerary carpal (3/7) and tarsal bones (2/7) (advanced ossification) were commonly found. However, two individuals (patients 2 and 4) had delayed carpal ossification which is quite contrary to what has been reported [1, 15]. We excluded hypothyroidism as the cause of delayed ossification in both of them.

Mother and son (patients 6 and 7) shared the same causative *FLNB* mutation, but showed some phenotypic differences (Table 1). Short stature and scoliosis were only present in the mother, whereas cervical instability was evident in the child and not in his mother. Although these clinical features are typically seen in subjects with LS, they are not obligatory present in all affected individuals and may develop with age. Such a high variability in severity of ph**enotypic expression in LS-affected family memb**ers carrying the same *FLNB* mutation has been reported by others [15, 16, 30, 31].

We identified a causative *FLNB* mutation in each of the five unrelated patients and the mother-son-duo with LS. All alterations are point mutations predicting an amino acid substitution. However, the c.2055G > C mutation turned out to affect splicing of the *FLNB* pre-mRNA (see below). One amino acid alteration is located in the Ig repeat 5, one in repeat 14 and four in repeat 15. Consistently, many LS-associated amino acid changes cluster in repeats 14 and 15 of the filamin B protein [15, 16, 24], including the most common *FLNB* mutation, p.(Gly1722Ser) (p.(Gly1691Ser) according to NM_001457.3), which has been identified in patient 2 of our cohort. This substitution has mainly been described in individuals with LS [1, 15, 16, 24], however, two subjects with a diagnosis of AOIII have also been reported to carry this mutation [4, 24]. Similarly, codons 1612 and 1643 (NM_001457.3) were found to be mutated in individuals with AOIII [p.(Gly1612Asp), p.(Ala1643Pro) and p.(Ala1643Ser)] [4, 24]. In patient 1 with LS of our cohort, we observed the novel substitution p.(Gly1612Ser) [p.(Gly1643Ser) according to NM_001164317.1], and the undescribed change p.(Ala1643Val) [p.(Ala1674Val) according to NM_001164317.1] was found in the mother-son-duo (patients 6 and 7). These findings further underscore that LS and AOIII have significant clinical overlap, both belonging to a spectrum of autosomal dominantly inherited skeletal phenotypes.

The *de novo* c.2055G > C transversion identified in patient 5 predicted the amino acid substitution from glutamine to histidine at position 685 in filamin B repeat 5. However, mRNA analysis revealed that the guanine-to-cytosine change at the last nucleotide of exon 13 causes loss of the adjacent splice donor site and usage of a cryptic splice donor in intron 13. The resulting *FLNB* transcript harbored the mutant base cytosine in the triplet 685 encoding histidine followed by nine additional *FLNB*-unrelated codons. Thus, the c.2055G > C alteration likely leads to the production of a filamin B protein lacking glutamine 685 but harboring ten novel amino acids between residues 684 and 686 (p.Gln685delins10). Remarkably, a similar *in-frame* insertion has been reported in an AOIII-affected individual: the nine amino acid insertion p.Gln685_Asp686ins9 was the consequence of the splice mutation c.2055 + 1G > A [24] with usage of the same cryptic splice site (at +28 and +29 in intron 13) reported here for the c.2055G > C mutation. The two splice mutations are the only known *FLNB* alterations that lead to an altered amino acid composition of the Ig repeat 5 associated with AOIII [24] and LS (this study). Taken together, all LS-associated *FLNB* mutations reported so far are predicted to preserve the reading frame and produce full-length filamin B which is in accordance with a proposed gain-of-function effect of the mutations leading to LS, AO and BD [24].

The pathogenic mechanisms underlying LS, AO and BD have not yet been fully explored. One cluster of disease-associated mutations is found in the N-terminal CH2 domain of filamin B [4, 15, 16, 24]. The two calponin homology domains (CH1 and CH2) constitute the actin-binding domain and regulate the binding of actin to filamins [32]. The mutations p.Trp148Arg and p.Met202Val have been demonstrated to confer enhanced actin binding of the filamin B mutant proteins, most likely by negatively affecting the CH2 regulatory function [27]. In line with this, expression of the mutant proteins FLNB^Ser235Pro^ and FLNB^Lys171Arg^ led to localization of the proteins within subcellular foci containing actin suggesting that these amino acid substitutions in the CH2 domain increase binding of filamin B to actin [24]. While the *FLNB* mutation p.Gly751Arg in repeat 6 also caused focal accumulation of the mutant protein, the p.Ser1602Pro substitution in repeat 14 and p.Pro1699Ser in repeat 15 did not [24]. Thus, although the Ig repeats 9 to 15 are necessary for high avidity F-actin binding [33], amino acid changes affecting these FLNB regions seem to act through yet unknown molecular mechanisms. Importantly, repeats 14 and 15 harbor another cluster of *FLNB* mutations linked to disease and lie adjacent to the flexible hinge 1 region which is central for the mechanosensory properties of filamins [34]. Indeed, filamins have been shown to act as mechanoprotective elements after force application and stabilize the plasma membrane [35, 36]. Thus, an increase in actin affinity and possible changes in mechanosensoring and/or mechanotransduction of filamin B imposed by mutations in the CH2 domain and repeats 14 and 15, respectively, may lead to the BD-AO-LS spectrum of diseases.

## Conclusions

Our study demonstrates that LS apparently has a homogeneous phenotype and shows allelic heterogeneity. We add five novel mutations, with four of them in the hotspots (Ig repeats 14 and 15) previously associated with LS. The c.2055G > C mutation was found to alter *FLNB* pre-mRNA splicing most likely giving rise to a filamin B protein lacking glutamine 685 and harboring ten FLNB-unrelated amino acids between residues 684 and 686. Thus, all reported *FLNB* mutations associated with LS leave the protein intact and likely confer gain-of-function. In summary, LS represents a clinically and radiographically characteristic disorder. A molecular diagnosis for LS can be achieved by testing selected exons of the *FLNB* gene, such as the previously reported exons 2–5 and 27–33 as well as exon 13 reported here.
